# Pathological and Ecological Host Consequences of Infection by an Introduced Fish Parasite

**DOI:** 10.1371/journal.pone.0026365

**Published:** 2011-10-17

**Authors:** J. Robert Britton, Josephine Pegg, Chris F. Williams

**Affiliations:** 1 Centre for Conservation Ecology and Environmental Change, School of Applied Sciences, Bournemouth University, Poole, Dorset, United Kingdom; 2 Environment Agency, Brampton, Cambridgeshire, United Kingdom; Institute of Marine Research, Norway

## Abstract

The infection consequences of the introduced cestode fish parasite *Bothriocephalus acheilognathi* were studied in a cohort of wild, young-of-the-year common carp *Cyprinus carpio* that lacked co-evolution with the parasite. Within the cohort, parasite prevalence was 42% and parasite burdens were up to 12% body weight. Pathological changes within the intestinal tract of parasitized carp included distension of the gut wall, epithelial compression and degeneration, pressure necrosis and varied inflammatory changes. These were most pronounced in regions containing the largest proportion of mature proglottids. Although the body lengths of parasitized and non-parasitized fish were not significantly different, parasitized fish were of lower body condition and reduced weight compared to non-parasitized conspecifics. Stable isotope analysis (δ^15^N and δ^13^C) revealed trophic impacts associated with infection, particularly for δ^15^N where values for parasitized fish were significantly reduced as their parasite burden increased. In a controlled aquarium environment where the fish were fed *ad libitum* on an identical food source, there was no significant difference in values of δ^15^N and δ^13^C between parasitized and non-parasitized fish. The growth consequences remained, however, with parasitized fish growing significantly slower than non-parasitized fish, with their feeding rate (items s^−1^) also significantly lower. Thus, infection by an introduced parasite had multiple pathological, ecological and trophic impacts on a host with no experience of the parasite.

## Introduction

Emerging infectious diseases are associated with pathogens that have recently increased in incidence, impact or geographic or host range [1], [2]. They may pose a greater threat to biodiversity through biomass loss and extinctions of host species than pathogens responsible for endemic diseases [3], [4]. This is because the dynamics of the host-parasite interactions may differ as the pathogen has not coevolved with the host or the ecosystem in which they emerged [5]. An outbreak of an emerging disease may occur when the parasitic fauna of a species is introduced from its natural range into a new region at the same time as its host, providing the opportunity for host switching [2], [6]. Given the lack of co-evolution between these new host species and the introduced pathogen, transmission rates and infection impacts may be high. Indeed, it has been suggested that the introduction of pathogens into new areas through human activities is one of the most important factors driving disease emergence in natural populations [2], [7].

Host responses to parasite infection are important to understand as they form the basis of the population response [8]. In co-evolved host: parasite relationships, infections tend to negatively impact host fitness, modulate the dynamics of host populations and have indirect consequences for non-host populations through changes in the strength of interspecific competitive relationships [9]. Infection costs are compensated by hosts through, for example, developing immune systems as an infection barrier and tolerance through alteration of life-history traits, particularly in the pre-reproductive life-span [10], [11]. This then impacts reproductive effort [12], [13] and body size [14] as individuals allocate more resources to reproduction than growth and survival to ensure reproduction before resource depletion, castration or death [15]. How hosts respond to introduced pathogens where there has been no co-evolution is less clear; whilst expectations are of catastrophic outcomes through strong negative effects on host survivorship [16], these may not always be apparent [17].

In freshwater ecosystems, the opportunity for fish parasites to be moved between regions by anthropogenic activities is high given that the rate of introduction of non-native fishes has doubled in the last 30 years, mainly due to the globalization of aquaculture [18]. Introduced diseases resulting through fish movements in aquaculture have already resulted in significant impacts in some fish, for example the infection of European eel *Anguilla anguilla* with *Anguillicoloides crassus* has been implicated as a major factor in their global decline [19], [20]. Research on the host consequences of infection by introduced parasites has tended to focus on those that impact immediately on host population dynamics through high mortality rates [6], with sub-lethal impacts, such as alterations in host behaviour and growth rates rarely considered despite their potentially significant consequences for wild populations [6], [17]. Consequently, the aim of this study was to identify the pathological, growth rate, feeding rate and trophic consequences of infection in a host: parasite relationship lacking co-evolution. Objectives were to use parasitized and non-parasitized fish sourced from a wild population that were free of confounding infections to identify the: (i) parasite prevalence and abundance within the host population; (ii) pathological consequences of the parasite in parasitized hosts; (iii) the consequences of infection for the weight and condition of hosts (as a proxy of life history traits generally); and (iv) whether parasitized hosts were impacted in their feeding (trophic niche). It was then identified whether any measured consequences of infection were reversible when the parasitized fish were held in captivity in a stable, non-competitive environment. It was predicted that due to the lack of experience of hosts with the parasite, the pathology of infection would be severe and be allied to the sub-lethal impacts of reduced growth and feeding rates, and occupying lower trophic levels compared to non-parasitized fish.

## Materials and Methods

### Ethics statement

All animal work was conducted in accordance to national and international guidelines to minimize discomfort to animals. All regulated procedures completed under the Animals (Scientific Procedures) Act 1986 were licensed by the UK Home Office under project licence number PPL 30/2626. The Ethics Review Panel of the School of Applied Sciences of Bournemouth University approved this project licence.

### Host:parasite model

The host: parasite model used was the fish common carp *Cyprinus carpio* and the intestinal fish parasite *Bothriocephalus acheilognathi*, a cestode native to Japan and China where it infects members of the Cyprinidae family [21], [22]. *Bothriocephalus acheilognathi* has been moved extensively throughout the global aquaculture trade via movements of grass carp *Ctenopharyngodon idella* and common carp and is now present across in every continent except Antarctica. It has low definitive-host specificity [23], [24], enabling it to infect a wide range of new hosts in its expanded range [25]–[27]. It has a simple two-host life cycle where free-living copepods are the intermediate host and fish the definitive host. Fish obtain infection by the ingestion of parasitized copepods [28]. *Cyprinus carpio* is an important aquaculture species world-wide, with global production in 2009 worth over US $4Billion [29]. It is also a highly prized angler-target species in many European countries [30]. It is, however, perceived as an invasive pest elsewhere [30].

In the study, parasitized and non-parasitized young-of-the-year *C. carpio* from a pond (1 ha in area) in Southern England were used as the model host as (i) *Bothriocephalus acheilognathi* is alien to England [31] and this *C. carpio* population was not known to have had any previous exposure to the parasite prior to its introduction to the pond in 2009; (ii) juvenile fishes are generally most vulnerable to infection by copepod intermediate hosts due to importance in diet [32]; (iii) losses through infection may yet to have been significant in the cohort, providing robust estimates of parasite prevalence and abundance; and (iv) impacts of parasites tend to be more severe in juvenile fishes compared with adults [17].

### Fish sampling and parasitological examinations

The fish were sampled from the pond in September 2010 using a micromesh seine net of 25 m length and 1.5 m depth. All captured *C. carpio* (parasitized/ non-parasitized) were removed and taken back to the laboratory. Of these, 70 were used for initial analysis. These were euthanized by lethal anaesthesia (5% w/v benzocaine) and the data collected from each fish being their length (fork, nearest mm), weight (nearest g), their infection status according to *B. acheilognathi* following dissection of their intestine, and the presence of any other parasitic infections (parasitized or non-parasitized). Of these other pathogens, only very light infections of common monogenea and protist parasites were recorded, but these were considered normal and insignificant (i.e. were not confounding infections). The fish data were then analysed for mean lengths (± standard deviation) of all, non-parasitized and parasitized fish, parasite prevalence (proportion of parasitized fish, %), parasite abundance (number of parasites per fish, weight of parasites per fish) and condition of individual fish (K, where K = 100×W/L^3^, where L was measured in cm). The somatic weight of parasitized fish was determined as total body weight minus parasite weight.

### Histopathological observations

Histopathology of the intestines was completed to compare the intestinal structure and morphology between parasitized with non-parasitized fish. Regions of the intestine were fixed in Bouins fixative for 24 hours before transferring to 70% Industrial Methylated Spirit. Tissues were trimmed, dehydrated in alcohol series, cleared and then embedded in paraffin wax. Sections of 3 to 4 µm were dried at 50°C, stained using Mayer's haematoxylin and eosin, and examined microscopically for pathological changes and described accordingly.

### Stable isotope analysis

A sample of dorsal muscle was taken from a random selection of individuals within the 70 individuals, with samples used from 17 parasitized and 17 non-parasitized fish in stable isotope analysis. This provided the values of δ^15^N (indicator of trophic level) and δ^13^C (indicator of energy source) [33] to reveal the extent of the trophic differences between the parasitized and non-parasitized fish. These data were complemented by the stable isotope analysis of the food items removed from the anterior third of their intestines. Due to their low sample weights, these food samples were combined within the parasitized and non-parasitized groups (6 samples per group). The use of food items in this part of the intestine was to minimise the chance of analysing food items that had already been heavily digested, although the taxonomic identification of these items could not be completed due to damage from the pharyngeal teeth of the fish. All samples were dried for 24 hours at 60°C before being processed at the Cornell Isotope Laboratory, Cornell University, New York, USA.

### Experimental observations of growth and feeding in live fish

Of the remaining *C. carpio* from the original sample captured from the wild, these were split into two groups, parasitized and non-parasitized, according to their gross morphology (parasitized fish tended to be thinner and with some abdominal distension due to the parasites) and presence of *B. acheilognathi* eggs in faeces. Infection was confirmed at the conclusion of all experiments through euthanasia and dissection of the intestine, with pre-dissection infection estimates of infection being 100% accurate. For the experiments, these fish were then transferred to tank aquaria (45 l tanks, flow through system) where they were used to determine their growth and feeding rate, and trophic position under controlled conditions. These trials were done in tandem, with 30 fish (n = 15 for both groups) being used for each.

To determine their growth and feeding rates, individual carp were measured and weighed, separated into randomised pairs (parasitized vs. parasitized (n = 5), parasitized vs. non-parasitized (n = 5) and non-parasitized vs. non-parasitized (n = 5)), and held at a constant 20°C under a light∶ dark cycle of 14∶ 10 hours for 60 days. Other than when being used in feeding rate trials, the fish were fed *ad libitum* daily using fish-meal based pellets used frequently in *C. carpio* aquaculture (2 mm diameter). The feeding rate trials were completed in a 24 day period within the growth trial. Feeding rate was measured through introducing the pellets into clean bottom tanks at densities of 20, 30, 40, 50, 70 and 100 items per tank (equivalent to 148, 222, 296, 370, 519 and 740 items m^−2^ respectively, where the mean dry weight of an individual of both items was 0.011 g) and filming the fish feeding response for 5 minutes. The response of all fishes to each food density was completed twice to provide replication. At the end of each filming, any uneaten food was removed immediately. In the video analysis, the data recorded was the time between the fish taking its first and fifth food item; feeding rate was then determined as the number of items taken per second. To identify the influence of infection on the feeding rate, ANCOVAs were used that enabled the effects of food density, fish starting length and the pairing of the fish (parasitized×parasitized, parasitized×non-parasitized, non-parasitized×parasitized) to be controlled in the models. At the end of the 60 day period, the fish were removed, re-measured and weighed. The growth rate of fish in the two groups was then determined though (i) specific growth rate (*SGR*; % d^−1^), calculated by [ln*W*_t+1_−ln*W*_t_]/t]×100, where *W*_t_ and *W*_t+1_ were the individual weights at the start and at the end of the period respectively, and *t* was the duration of the experimental period (60 days); and (ii) incremental fork length (*IL*; mm d^−1^), calculated by [*L*_t+1_−*L*_t_]/t, where *L*_t_ and *L*_t+1_ and *t* were as per specific growth rate (substituting *L* for *W*).

The 30 fish used for analysis of their trophic position were held under the same husbandry conditions as the fish used for feeding and growth rate. These fish were however, fed *ad libitum* daily for 120 days to allow for assimilation and turnover of their new food sources into their tissues. After the end of the period, the fish were removed, euthanized, muscle samples taken for stable isotope analysis, with these complemented by analysis of pellet samples (n = 6 samples for analysis). These were analysed as per those already described for stable isotope analysis.

### Statistical analyses

Data to determine if differences in the length, weight, condition, feeding rate and the stable isotope values of δ^15^N and δ^13^C between parasitized and non-parasitized fish were significant in both wild and captive conditions were initially tested for normality and log transformed where necessary. Parametric tests were then used to test for significant differences in mean values using ANOVA; ANCOVA was used where covariates had to be controlled in the analyses, such as the allometric effect of body length. The ANCOVA models were only considered valid and used subsequently when the assumptions were met that variances were equal between the groups (Levene's test, *P*>0.05), there was no interaction between the covariates and the groups (homogeneity of the regression slope; *P*>0.05) and where the test results were significant, post-hoc power analysis indicated statistical power>0.80. Where error is provided around mean values, they represent 95% confidence limits unless stated otherwise. All statistical tests were completed in SPSS v. 16.0 and assessed at α = 0.05.

## Results

### Parasitized vs. non-parasitized fish in the wild

The mean length and weight of the 70 *C. carpio* analysed initially in the laboratory was 29.2±4.3 mm and 0.49±0.21 g respectively; of these, 32 were parasitized with *B. acheilognathi* (parasite prevalence 45.7%). Parasite abundance ranged between 1 and 7 cestodes in the intestine (mean 2.0±1.7 cestodes), with their total weight per fish (burden) ranging between 0.004 and 0.069 g (mean 0.015±0.016 g) which comprised between 0.56 and 11.95% of fish total body weight (mean 2.98±3.31%). The relationship between cestode burden and fish length was not significant (R^2^ = 0.11; F_1,30_ = 0.93, *P*>0.05). There was no significant difference in the mean lengths of parasitized (29.9±4.5 mm) and non-parasitized fish (28.7±4.1 mm) (ANOVA F_1,68_ = 0.83, *P*>0.05). There was, however, a significant difference in the condition of parasitized (0.017±0.0018) fish compared with non-parasitized fish (0.019±.00019) (ANOVA F_1,68_ = 21.28, *P*<0.01). Comparing the somatic weight of parasitized and non-parasitized fish whilst controlling for the effect of fish length revealed parasitized fish weighed significantly less than the non-parasitized fish (Table 1).

**Table 1:** Infection effects of *Bothriocephalus acheilognathi* on the weight of *Cyprinus carpio* captured from the wild.

Effect		Weight (g)
Infection		F_1,65_ = 20.36, *P*<0.01
Fish length		F_1,65_ = 943.7, *P*<0.01
Difference between groups (mean ± S.E.)		
Non-parasitized	Parasitized	0.10±0.02 *P*<0.01

Fish length was the covariate in the ANCOVA model; corresponding differences between parasitized and non-parasitized fish are indicated by pairwise comparisons with Bonferroni adjustments for multiple comparisons.

**Figure 1:**
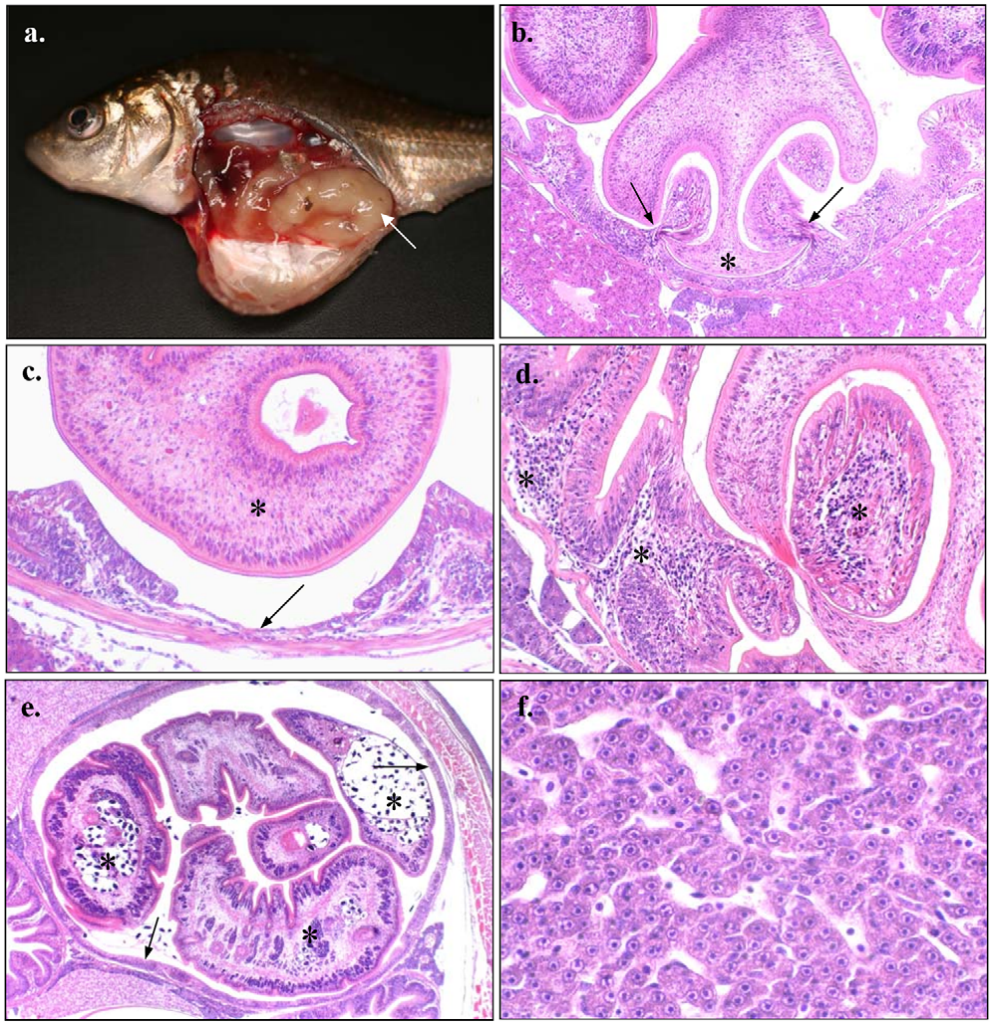
Histopathology of *Cyprinus carpio* intestines infected with *Bothriopcephalus acheilognathi*. (a) Young-of-the-year *Cyprinus carpio* parasitized with *Bothriopcephalus acheilognathi* showing enlarged, transparent and occluded intestine (arrow); (b) attachment of *B. acheilognathi* involving the two bothria located on each side of the scolex pinching the intestinal folds (arrow). This forced the apex of the scolex (*) firmly against the gut wall; (c) pressure exerted by the scolex (*) created indentations within the intestine, with loss of mucosa and pronounced thinning of the gut wall (arrow); (d) marked inflammatory response to *B. acheilognathi* infection with an increase in lymphocytes throughout the lamina propria (*); (e) *B. acheilognathi* proglottids (*) within the anterior intestine of common carp, causing pronounced intestinal distension, compression of intestinal folds (arrows) and extensive loss of normal gut architecture; and (f) Atrophy of hepatocytes throughout the liver of parasitized common carp, consistent with reduced nutritional status.

All of the parasitized fish revealed evidence of gross pathological changes in the intestines due to the parasite(s) (Figure 1). The intestinal tracts of these hosts were enlarged, thin-walled and occluded (Figure 1a), and in many fish, the intestinal wall was sufficiently stretched to be transparent, allowing the individual body segments (proglottids) of *B. acheilognathi* to be seen within the gut lumen. The cestodes were attached to the intestine in the region posterior to the bile duct opening, primarily occupying the anterior third of the intestine, although proglottids also extended into the lower half of the tract. Pathological changes to the intestinal tract could be broadly divided into those caused by scolex attachment and those associated with the body of the cestodes. During attachment, the bothria (located on each side of the scolex) were used to engulf the intestinal folds (Figure 1b). This provided a firm site of attachment, forcing the apex of the scolex against the gut wall (Figure 1b). This led to compression and thinning of the intestine, and formation of localised pits that extended as far as the muscularis (Figure 1c). Pinching of intestine between the bothria caused focal haemorrhaging, localised necrosis of the mucosa and an increase in the number of lymphocytes within the lamina propria (Figure 1d). This was often accompanied by the loss and degeneration of epithelium. Pathological changes were most extensive in regions containing the largest proportion of mature proglottids (Figure 1e). These regions were characterised by intestinal occlusion, mechanical distension and compression of the intestinal folds (Figure 1e), with congestion, pressure necrosis and atrophy of the mucosa. Heavy parasite burdens led to a complete loss of normal gut architecture within affected regions. The severity of these changes was associated with total cestode mass rather than the number of individual parasites. Histopathological observations of the liver revealed atrophy of hepatocytes in a number of the heavily parasitized fish (Figure 1f).

**Figure 2:**
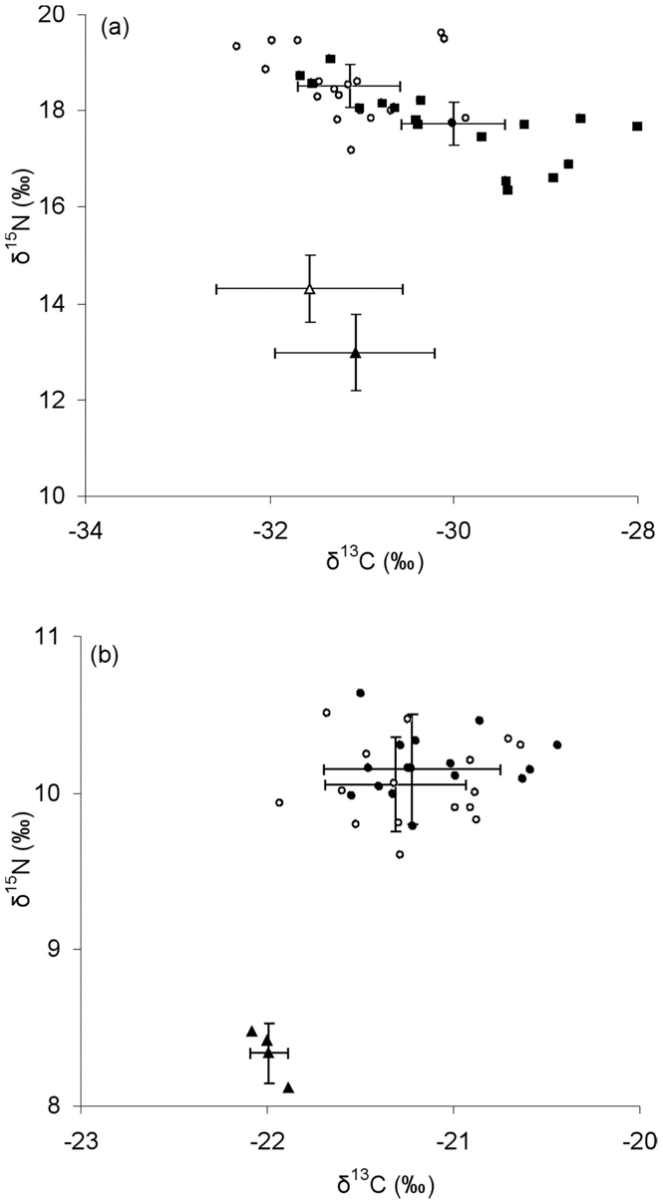
Stable isotope biplots of *Cyprinus carpio* parasitized (•) and non-parasitized (○) with *Bothriocephalus acheilognathi* (means and 95% confidence limits shown). (a) the cohort of young-of-the-year *Cyprinus carpio* from the wild and (b) fish from the same population but held in captivity and fed ad libitum for 120 days. Values are estimated marginal means from ANCOVA where the effect of fish length was controlled (c.f. Table 4, 5). Also shown are the mean values of (a) food items in the stomach contents of parasitized (▴) and non-parasitized fish (▵), and (b) the fish meal pellets fed *ad libitum* to the captive fish (▴). Note the different values between (a) and (b) used on the x- and y-axes.

**Figure 3:**
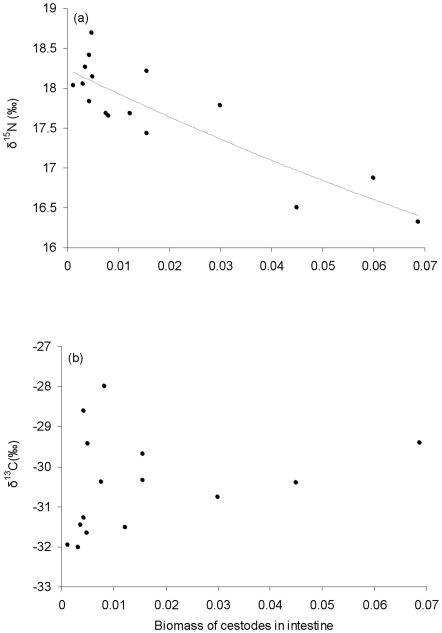
Relationship of (a) δ^15^N and (b) δ^13^C and parasite burden in *Cyprinus carpio* parasitized with *Bothriocephalus acheilognathi*. The solid line represents the significant relationship between δ^15^N and parasite burden according to polynomial regression (*cf.* Results).

Stable isotope analysis revealed that the parasitized fish had mean values of δ^15^N and δ^13^C that were significantly different from non-parasitized fish (Table 2; Figure 2). This was also reflected in significant differences in the δ^15^N and δ^13^C of the food items in the anterior third of their intestines (items in parasitized vs. non-parasitized fish: mean δ^15^N 12.98±0.81, 14.31±0.69, ANOVA F_1,4_ = 5.11, *P*<0.05; δ^13^C −31.07±0.87, −31.56±0.91, ANOVA F_1,4_ = 4.11, *P*<0.05). For parasitized fish, increased parasite burdens were significantly associated with reduced values of δ^15^N (polynomial regression: R^2^ = 0.75; F_2,13_ = 19.90, *P*<0.01), but this was not apparent for δ^13^C (linear regression R^2^ = 0.15; F_1,14_ = 2.44, *P*>0.05) (Figure 3).

**Table 2:** Stable isotope analysis of *Cyprinus carpio* parasitized by *Bothriocephalus acheilognathi*.

(a) δ^15^N		
Effect		Mean δ^15^N
Group (infection/ not parasitized)		F_1,31_ = 2.70, *P*<0.05
Fish length		F_1,31_ = 18.64, *P*<0.01
Group × fish length		F_1,31_ = 1.59, *P*>0.05
Difference between treatments (mean ± S.E.)		
Not parasitized	Parasitized	0.79±0.24‰, *P*<0.05

(b) δ^13^C		
Effect		Mean δ^13^C
Infection/ not parasitized		F_1,31_ = 4.70, *P*<0.05
Fish length		F_1,31_ = 21.63, *P*<0.01
Group × fish length		F_1,31_ = 1.59, *P*>0.05
Difference between treatments (mean ± S.E.)		
Not parasitized	Parasitized	−0.91±0.29‰, *P*<0.05

ANCOVA outputs comparing (a) δ^15^N and (b) δ^13^C for the natural population of young-of-the-year *C. carpio* consisting of two groups: (i) fish parasitized with *B. acheilognathi* and (ii) not parasitized. Corresponding differences between groups, indicated by pairwise comparisons with Bonferroni adjustments for multiple comparisons, are shown.

### Parasitized vs. non-parasitized fish in controlled conditions

When the 30 parasitized (n = 15) and non-parasitized (n = 15) fish were held in tanks for 60 days at a constant 20°C and fed *ad libitum* with fish-meal pellets, the parasitized *C. carpio* grew significantly slower than their non-parasitized conspecifics in both length and weight (Table 3). Although all fish had equal access to the food resources, the feeding rate (items s^−1^) of non-parasitized fish was significantly higher than for parasitized fish, irrespective of the infection status of their paired conspecific (Table 4). Of the final 30 *C. carpio* held for 120 days at a constant 20°C and fed *ad libitum* daily with fish-meal pellets, there were no significant differences in the mean δ^15^N and δ^13^C values for parasitized and non-parasitized *C. carpio* at the end of the period (Table 5, Figure 2).

**Table 3:** Infection effects of *Bothriocephalus acheilognathi* on the growth of *Cyprinus carpio*.

(a)		
Effect		Specific growth rate (% d^−1^)
Infection		F_1,27_ = 4.88, *P*<0.05
Fish weight		F_1,27_ = 6.01, *P*<0.02
Pairing of parasitized/ non-parasitized fish		F_1,27_ = 0.98, *P*>0.05
Difference between groups (mean ± S.E.)		
Non-parasitized	Parasitized	0.64±0.29, *P*<0.05

(b)		
Effect		Incremental fork length (mm d^−1^)
Infection		F_1,27_ = 6.43, *P*<0.02
Fish length		F_1,27_ = 0.18, *P*>0.05
Pairing of parasitized/ non-parasitized fish		F_1,27_ = 1.06, *P*>0.05
Difference between groups (mean ± S.E.)		
Non-parasitized	Parasitized	0.11±0.04, *P*<0.02

ANCOVA outputs of (a) specific growth rate and (b) incremental fork length of *C. carpio* held and fed *ad libitum* for 60 days at 20°C. Starting fish weight and length were the respective covariates in the ANCOVA model; corresponding differences between parasitized and non-parasitized fish are indicated by pairwise comparisons with Bonferroni adjustments for multiple comparisons.

**Table 4:** Infection effects of *Bothriocephalus acheilognathi* on the feeding rate (items s^−1^) of *Cyprinus carpio*.

Effect		Feeding rate
Infection		F_1,237_ = 7.41, *P*<0.01
Food density		F_1,237_ = 11.77, *P*<0.01
Individual fish		F_1,237_ = 2.09, *P*>0.05
Fish length		F_1,237_ = 0.84, *P*>0.05
Pairing of parasitized/ non-parasitized fish		F_1,237_ = 0.73, *P*>0.05
Difference between groups (mean ± S.E.)		
Non-parasitized	Parasitized	0.55±0.20, *P*<0.01

Fish length, number of food items released and individual fish were the covariates in the ANCOVA model; corresponding differences between parasitized and non-parasitized fish are indicated by pairwise comparisons with Bonferroni adjustments for multiple comparisons.

**Table 5:** Stable isotope analysis of *Cyprinus carpio* infected with *Bothriocephalus acheilognathi* and held in controlled conditions.

(a) δ^15^N		
Effect		Mean δ^15^N
Group (infection/ not parasitized)		F_1,27_ = 0.05, *P*>0.05
Fish length		F_1,27_ = 2.01, *P*>0.05
Group × fish length		F_1,27_ = 1.58, *P*>0.05
Difference between treatments (mean ± S.E.)		
Not parasitized	Parasitized	0.04±0.18‰, , *P*>0.05

(b) δ^13^C		
Effect		Mean δ^13^C
Infection/ not parasitized		F_1,27_ = 3.42, *P*>0.05
Fish length		F_1,27_ = 1.94, *P*>0.05
Group × fish length		F_1,27_ = 1.58, *P*>0.05
Difference between treatments (mean ± S.E.)		
Not parasitized	Parasitized	0.29±0.28‰, *P*>0.05

ANCOVA outputs comparing (a) δ^15^N and (b) δ^13^C for young-of-the year *Cyprinus carpio* held in captivity for 120 days and fed *ad libitum* on fish-meal pellets, where the fish consisted of two groups: fish parasitized with *Bothriocephalus acheilognathi* and not parasitized. Corresponding differences between groups, indicated by pairwise comparisons with Bonferroni adjustments for multiple comparisons, are shown. *NS* = not significant; *P<0.05, **P<0.01.

## Discussion

The growth and condition of *C. carpio* parasitized with *B. acheilognathi* was compromised, with wild fish and those subsequently held in controlled conditions being of reduced condition and slower growing when compared with non-parasitized conspecifics. This was consistent with both the pathological damage to the intestinal tract of parasitized fish that was likely to have impaired its normal function and the reduced feeding rate of hosts that appeared to impede their ability to capture similar food items as non-parasitized fish in the wild. This was reflected in their diet that generally comprised items of lower trophic status (δ^15^N). Whilst this trophic difference was reversible in controlled conditions when the fish were fed identical diets, their growth remained impaired, suggesting a range of infection consequences for hosts. In addition to these measured effects of parasitism by *B. acheilognathi*, it was likely that the infected fish had other physiological disturbances that could not be measured here. In entirety, it would be questionable as to whether these compromised hosts could have survived a winter period due to their poor condition, emphasizing that sub-lethal infection consequences may eventually lead to lethal ones. These findings were consistent with the prediction that hosts would be severely compromised by infection due to their lack of previous experience with the parasite.

The relationship of host growth with infection can be complex when parasites are trophically transmitted, as individual fish that initially have high ingestion rates and so faster growing may be more vulnerable to eating parasitized intermediate hosts, with infection only then reducing the host growth rate [34]. Here, however, there was no evidence to suggest faster growing individuals were those that became parasitized. Given that the parasitized *C. carpio* were young-of-the-year and of small body size (<35 mm at the commencement of the study), then their reduced growth may be more related to their intolerance of infection rather than reflecting a change in resource allocation to ensure reproduction prior to death [10], [15]. In some cestode parasites, increased host growth is observed following infection, such as in those that castrate their hosts that enable increased energy allocation to somatic growth [14], [35], [36]. Whilst this was not evident here, immature hosts were being studied, with *C. carpio* usually maturing at lengths of at least 110 mm and often during their third year of life [37]. Nevertheless, the findings in this study were consistent with other studies on impacts of *B. acheilognathi* on naïve hosts that suggest detrimental effects including intestinal abrasion and disintegration, blockage and perforation of the intestinal tract, emaciation and anemia in chronic infections, decrease in intestinal, hepatic and pancreatic enzymes, and decrease in haemoglobin content [26], [38]–[40].

Whilst native parasites are increasingly recognized as important in controlling host populations and the structure of communities [41], [42], how introduced parasites may control host populations is unclear. Studies on captive fishes parasitized with introduced parasites such as *B. acheilognathi* usually report very high mortality rates [28]. Although these studies may be of limited utility to wild populations given that in aquaculture high transmission rates and virulence may occur through unnaturally high stock densities [43], wild studies on other naïve host: introduced parasite systems have suggested similar consequences. For example, the effects of the introduced parasitic fly *Philornis downsi* on the ground finch *Geospiza fortis* in the Galápagos Islands was to significantly increase morality rates [44], [45]. Across Europe, dramatic population declines have occurred in natural populations of native crayfish (e.g. *Astacus astacus*), oysters (*Ostrea edulis*) and eels that can all be attributed, in varying degrees, to introduced pathogens and the lack of immunity in the naïve hosts through the lack of co-evolution [2]. This co-evolution between host and parasite is thus important in building resistance and tolerance of the host to infection, especially as this has a strong genetic component, particularly through genes in the MHC (major histocompatibility) complex [46]. Moreover, moderately heterozygous fish have been shown to be less resistant and tolerant to infection by an ectoparasite than those that were highly heterozygous or homozygous [47], with rapid evolution in response to parasitism demonstrated in a range of freshwater host species, including some fishes, that appears to have protected some populations from the virulent effects [16].

In summary, in this host-parasite system lacking co-evolutionary processes, pathological impacts on parasitized hosts were marked, with additional trophic consequences that resulted in reduced growth and condition of individuals even in a controlled environment and *ad libitum* feeding. This is consistent with findings in other host: parasite relationships where there has been no co-evolution that suggest catastrophic impacts can occur in the host population [16]. In the case of *C. carpio*, their global importance to aquaculture [29] and recreational fisheries [30] suggest that in addition to the infection consequences outlined here, then there may also be important socio-economic impacts to consider.
